# Influence of Cyanide and Thiocyanate on the Formation of Magnetite Synthesized under Prebiotic Chemistry Conditions: Interplay between Surface, Structural, and Magnetic Properties

**DOI:** 10.1021/acsomega.4c11450

**Published:** 2025-03-25

**Authors:** Rafael Block Samulewski, Ismael Leandro Graff, Slavomír Nemšák, Dimas Augusto Morozin Zaia

**Affiliations:** † Programa de Pós-Graduação em Ciência e Engenharia de Materiais (PPGCEM), Universidade Tecnológica Federal do Paraná UTFPR, Apucarana, Paraná CEP 86812-460, Brazil; ‡ Departamento de Física, Universidade Federal do Paraná UFPR, Curitiba, Paraná CEP 81531-980, Brazil; § Advanced Light Source, Lawrence Berkeley National Laboratory, Berkeley, California 94720, United States; ∥ Departamento de Química, Universidade Estadual de Londrina, CEP Londrina, Paraná UEL 86057-970, Brazil

## Abstract

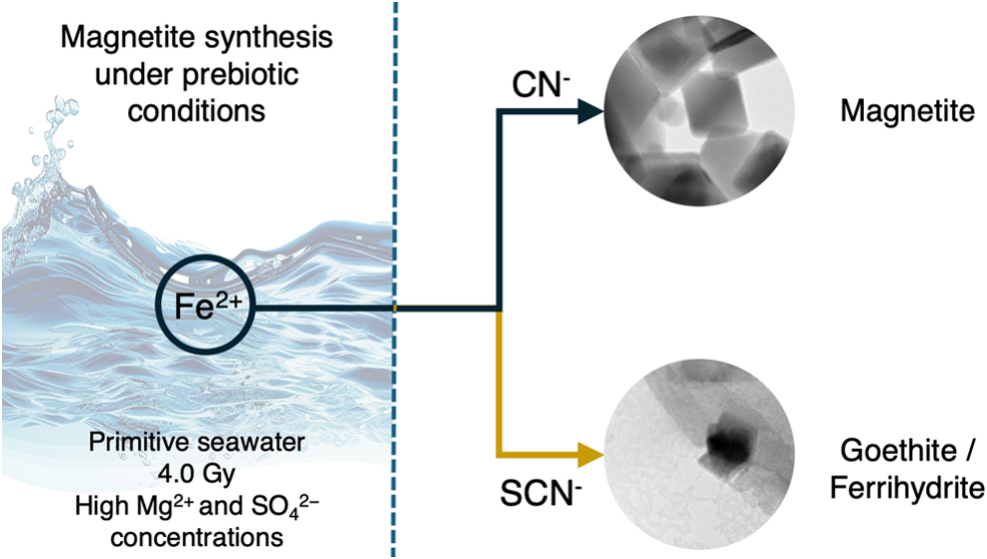

Understanding the chemical and geological conditions of early Earth is crucial to unraveling the processes that led to the evolution of life. Iron, abundant in the early oceans, likely played a significant role in the evolution of life, particularly in the form of minerals that supported the emergence of the first life forms. This article investigates the catalytic effects of cyanide and thiocyanate ions on magnetite samples synthesized under conditions that simulate the early Earth. Magnetite samples were characterized using X-ray photoelectron spectroscopy (XPS), Fe L_23_ near-edge X-ray absorption fine structure (NEXAFS), transmission electron microscopy (TEM), and magnetization measurements. The results reveal variations in elemental composition influenced by synthesis conditions, with cyanide ions promoting the formation of magnetite and seawater and thiocyanate inducing the formation of ferrihydrite and goethite, respectively, along with magnetite. These discoveries enrich our understanding of Earth’s earliest geochemical processes, contribute to new material synthesis routes, and help environmental science.

## 1 Introduction

Understanding the early Earth’s chemical and geological conditions is essential to elucidate the fundamental processes that contributed to the early evolution of life. A crucial aspect of these conditions is the composition of the atmosphere and primordial oceans, which play a crucial role in forming and stabilizing vital compounds and minerals.^1−5^

Iron emerges as a significant protagonist among the key elements present in this initial environment.^5−7^ The abundance of iron in the primitive oceans, mainly in the form of iron(II), was influenced by the reducing nature of the primordial atmosphere, which did not favor the oxidation of this metal.^8,9^ However, some recent studies have revealed that specific ions present in primitive seawater could have induced the formation of mineral phases containing oxidized iron, particularly iron(III). According to Bernal, it is very likely that minerals formed on primitive Earth played a key role in the emergence of life on Earth, and due to their high concentration, it has been argued that iron minerals might have played this role.^10,11^

In this context, the investigation of the synthesis of magnetite, a mineral form of iron oxide highly relevant in geosciences and biology, becomes fundamental.^12−14^ Magnetite (Fe_3_O_4_) is known for its magnetic properties, and its presence is commonly observed in varied geological environments. It is frequently formed in magmatic and hydrothermal environments from the oxidation of iron(II) ions.^15^ Since hydrothermal vents were more common in the prebiotic Earth than nowadays,^16^ magnetite could be considered volumetrically significant for prebiotic chemistry experiments.^17,18^

Cyanide and thiocyanate ions are considered very relevant as building blocks for the formation of amino acids. Consequently, they may have had an important role in biogenesis.^19−21^ They could have been delivered to the Earth by comets^22,23^ and could be synthesized in the atmosphere, by lightning and near hydrothermal vents.^24−26^ Thiocyanate has been artificially synthesized in experiments simulating prebiotic environments,^27−29^ as well as, it was found in hydrothermal vents and interstellar medium.^30,31^ Since cyanide, thiocyanate, and magnetite probably coexisted in the prebiotic Earth, it is very important to comprehend their interaction to prebiotic chemistry. Understanding their interaction is very important in studies of prebiotic chemistry under the specific conditions of early Earth offers valuable insights into the geochemical and biochemical processes that shaped the earliest stages of terrestrial evolution.

This article seeks to continue the investigation on the properties of magnetite synthesized under conditions that simulate the early Earth started earlier,^12^ considering not only the presence of iron(II) in the oceans but also the catalytic effects of specific ions in early seawater that favor oxidation of iron and the subsequent formation of mineral phases containing iron(III). Special emphasis is given to the study of the chemistry of the samples’ outer surface by making use of XPS and NEXAFS measurements. It is the surface that first reacts with the chemical environment, inducing the formation of new molecules or compounds. Data reveal that the addition of cyanide favors the formation of magnetite and, at the same time, acts as a protective agent against the oxidation of Fe^2+^ ions. Added thiocyanate ions, in turn, lead to the formation of iron(III)-pure oxides.^32,33^ By understanding the mechanisms underlying this process, we can shed light on the primordial geochemical events that gave rise to the conditions that led to the emergence of life on Earth and elucidate the formation of minerals composed of oxidized iron.

## 2 Experimental Section

### 2.1 Synthesis

Magnetite samples were prepared according to the methodology described by Samulewski et al.^12^ All solutions were prepared under a nitrogen-inert atmosphere. For the synthesis of the standard magnetite samples (MGP), the first solution (Solution 1) was prepared by dissolving 5.72 g (30 mmol) of ferrous chloride heptahydrate (FeCl_2_.7H_2_O) in 60 mL of previously deaerated ultrapure water, which was then heated to 90 °C. Simultaneously, a second solution (Solution 2) was prepared by dissolving 4.49 g (80 mmol) of potassium hydroxide (KOH) and 0.646 g (6.5 mmol) of potassium nitrate (KNO_3_) in 25 mL of deaerated ultrapure water, also heated to 90 °C. The pH of Solution 1 was adjusted to values compatible with estimates of the composition of primitive oceans, ranging from 6.0 to 7.5, based on geochemical simulation studies.^14,34,47^ Once both solutions reached the desired temperature, Solution 2 was slowly added to Solution 1, with a rate of approximately 4 mL per minute, while maintaining constant stirring until a dark precipitate formed. The resulting dispersion was stirred 40 min after the complete addition under an inert atmosphere. Subsequently, the dispersion was transferred to a refrigeration unit set at 5 °C and left undisturbed for 24 h. After this period, the black solid was filtered, washed three times with ultrapure water until the pH became stable at values between 6.0 and 7.5, and then subjected to freezing. Finally, the solid was lyophilized to obtain the dry product. Modifications to the methodology primarily focused on alterations in Solution 1 to prevent the precipitation of calcium and magnesium, anticipated due to the presence of potassium hydroxide in Solution 2.

Additionally, changes in the solution composition included the introduction of cyanide or thiocyanate ions and the substitution of ultrapure water with water that mimics seawater from 4.0 billion years ago (4.0 Gy), following the method described by Zaia et al.^14,47^ Samples are named according to synthesis details as follows: MGP for magnetite synthesized in ultrapure water; MG4P for magnetite synthesized in seawater 4.0 Gy; MGCN for magnetite synthesized in ultrapure water with added cyanide; MG4CN for magnetite synthesized in seawater 4.0 Gy with added cyanide; MGSCN for magnetite synthesized in ultrapure water with added thiocyanide, and MG4SCN for magnetite synthesized in seawater 4.0 Gy with added thiocyanide. Table 1 summarizes the information on the synthesis of the samples.

**Table 1:** Summary of Experimental Conditions for Magnetite Sample Synthesis^a^

sample	solution	cyanide	thiocyanate
MGP	ultrapure water	no	no
MG4P	seawater 4.0 Gy^a^	no	no
MGCN	ultrapure water	60 mmol	no
MG4CN	seawater 4.0 Gy^a^	60 mmol	no
MGSCN	ultrapure water	no	60 mmol
MG4SCN	seawater 4.0 Gy^a^	no	60 mmol

a 60 mL solution of seawater 4.0 Gy composition (mg): Na_2_SO_4_ (16.2); MgCl_2_·6H_2_O (30.0); CaCl_2_·2H_2_O (150.0); KBr (3.0); K_2_SO_4_ (24.0); MgSO_4_ (900.0).12,44 CN-potassium cyanide, SCN-potassium thiocyanate. Each synthesis was performed at least six times. All syntheses were performed with deaerated solutions.

### 2.2 Physical Measurements

Room-temperature X-ray photoelectron spectroscopy (XPS) measurements were performed at the 9.3.2 beamline of the Advanced Light Source (ALS), Lawrence Berkeley National Laboratory (LBNL).^35−37^ The soft X-ray ambient pressure (S-APXPS) end station is equipped with a Scienta R4000 HiPP hemispherical electron energy analyzer. An incident photon energy of 650 eV was chosen to reach the core levels of interest and to avoid Auger peaks overlapping photoelectron peaks. Besides, the photon flux is around its maximum for the beamline at this energy. With this incident photon energy and pass energy of 100 eV the energy resolution is ∼ 0.9 eV, obtained by measuring the full width at half-maximum (fwhm) of Au 4f_7/2_ peak of a gold foil fixed on the sample holder. Charge referencing was done by fitting C 1s peak of adventitious carbon (AdC) from the samples and considering the binding energy of C–C/C–H as 284.8 eV.^38,39^ Although critics regarding the use of AdC for charge referencing have been made in recent years,^40,41^ it has also been shown that if one understands the limits and possible issues with the use of AdC for charge referencing, reliable and meaningful results can be obtained.^42^ Several authors demonstrated that O 1s peak position virtually did not change for different iron oxides and suggested its use for charge referencing.^43−45^ In our case, we decided to use AdC for charge referencing since the O 1s peak region contains several convoluted peaks originating from (apart from the oxide lattice oxygen Fe–O) different functionalities (Ex.: OH, C–O–C, C = O, O–C = O), which imposes difficulties in the peak fitting process and introduce uncertainties. Though the C 1s peak also contains these same functionalities, its peak fitting became much more straightforward and less prone to ambiguities. Room-temperature Fe L_23_-edge Near-edge X-ray Absorption Fine Structure (NEXAFS) measurements in total electron yield (TEY) mode were also performed in the same setup. XPS and NEXAFS (in TEY mode) are both surface probes, but they access complementary aspects of the atoms’ environment and electronic structure.

The magnetite samples magnetization was evaluated through room-temperature *M* × *H* curves using a homemade vibration sample magnetometer (VSM), applying magnetic fields of up to 15 kOe. Transmission electron microscopy (TEM) images were obtained in a JEOL JEM-2100 equipment at an accelerating voltage of 200 kV. Samples were deposited on copper grids coated with an ultrathin carbon film (TedPella) by dispersing 3 μL of suspension diluted in water.

## 3 Results and Discussion

### 3.1 X-ray Photoelectron Spectroscopy (XPS)

**Figure 1:**
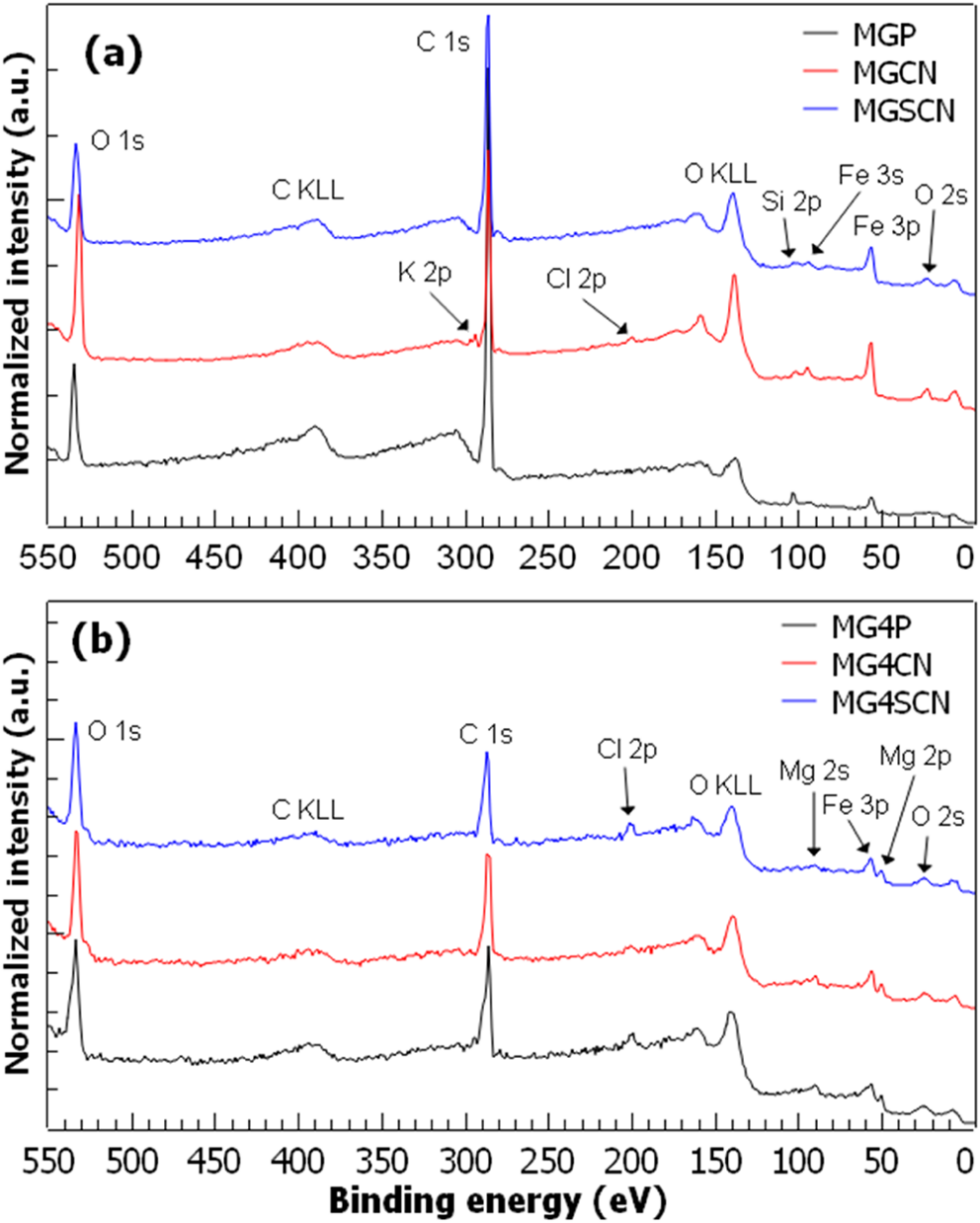
XPS survey scans for samples synthesized in (a) ultrapure water (MGP, MGCN, MGSCN) and (b) in seawater 4.0 Gy (MG4P, MG4CN, MG4SCN). MGP/MG4P: samples prepared without any additive. MGCN/MG4CN: samples prepared in the presence of potassium cyanide (60 mmol of KCN). MGSCN/MG4SCN: samples prepared in the presence of potassium thiocyanate (60 mmol of KSCN). The seawater 4.0 Gy (high Mg^2+^and SO_4_^2–^ concentrations) was prepared as suggested by Zaia.^47^ The pass energy used for the survey scans was 200 eV. For details of the preparation method, refer to Samulewski et al.^12^ Spectra were vertically shifted for comparison purposes.

Figure 1 shows the survey spectra of samples synthesized in (a) ultrapure water (MGP) and (b) in seawater 4.0 Gy (MG4P).^12,45−46,47^ Peaks identification was accomplished by making use of well-established databases.^48,49^ All spectra present peaks of O 1s, C 1s, and Fe 3p and Auger peaks of carbon and oxygen (C KLL and O KLL, respectively). The Auger peaks are distinguishable from the photoelectron peaks due to their broader and asymmetric structure, and their energy positions were confirmed by simulations using the SESSA code.^50^ In Figure 1a, a peak at around 102.0 eV is also observed and is attributed to Si 2p (SiO_*x*_), which probably comes from glassware. For samples MGCN and MGSCN, one identifies Fe 3s and O 2s peaks, and specifically for MGCN, Cl 2p is observed. Chlorine is present in one of the reagents used to synthesize the samples; ferrous chloride (FeCl_2_·7H_2_O). For the spectrum of sample MGCN, a peak on the high-BE side of C 1s is also observed, referring to K 2p. Table 2 presents a summary of the energy positions of all peaks identified in the survey scans. In the case of sample MGP, the intensity of the signal coming from C is quite large, while the one from Fe is relatively weak. Adding KCN in the synthesis process leads to a significant increase in the Fe and O signals concurrently with a decrease of the signal from C. It suggests that adding KCN favors the formation of iron oxide (or oxyhydroxide) and somehow protects the outer surface from organic species. Figure 1b shows the survey spectra of samples synthesized in seawater 4.0 Gy. Compared to Figure 1a, we note several differences: (i) the relative intensity of the carbon peak is much smaller; (ii) chlorine is identified in all samples; (iii) magnesium (Mg 2p, Mg 2s) is present; (iv) silicon is not detected. Magnesium comes from the seawater 4.0 Gy used to synthesize the samples since Mg^2+^ is the most abundant cation in its composition. Although calcium (Ca^2+^) is also plentiful in seawater 4.0 Gy it was not detected in XPS even though a gypsum phase (CaSO_4_·2H_2_O) was identified in X-ray diffraction (XRD) measurements for sample MG4SCN.^12^

**Table 2:** Binding Energies (*E*_B_) of All Elements Identified in the XPS Survey Scans^a^

Element	O	Mg	Fe	Mg	Fe	Si	O	Cl	C	K	C	O
Orbital	2s	2p	3p	2s	3s	2p	KLL	2p	1s	2p	KLL	1s
*E*_B_ (eV)	23.0	50.7	56.8	89.2	94.8	102.0	138.8	200.0	286	293.8	390	533.8

a These values refer to the center of the peaks and are meant to be a guide for the identification of each peak. The KLL label identifies Auger peaks.

**Figure 2:**
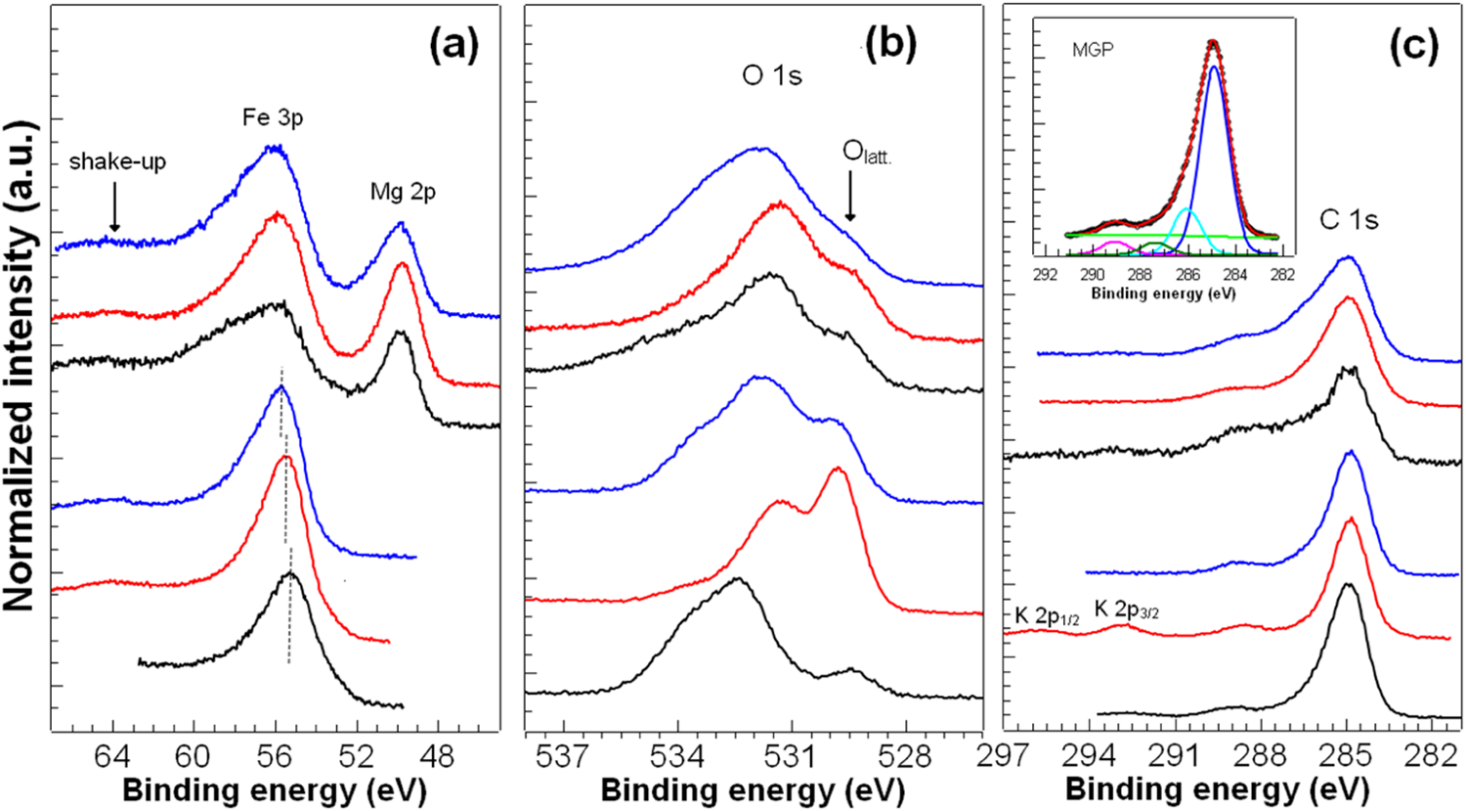
XPS measurements around (a) Fe 3p, (b) O 1s and (c) C 1s. From the bottom to the top, the first three curves refer to samples synthesized in ultrapure water and the next three curves for samples synthesized in seawater 4.0 Gy, following the same color-coding of Figure 1. Solid-black: MGP/MG4P. Solid-red: MGCN/MG4CN. Solid-blue: MGSCN/MG4SCN. The synthesis of MGCN/MG4CN and MGSCN/MG4SCN was carried out in the presence of 60 mmol of KCN and 60 mmol of KSCN, respectively. The inset in (c) shows the fitting for the MGP curve as an example of the fitting procedure, where the filled black circles represent the actual data, the red curve refers to the total fit, and the green curve to the Shirley-type background. The different functionalities are represented by the blue peak (C–C/C–H), the cyan peak (C–OH/C–O–C), the olive peak (C = O), and the magenta peak (O–C = O). Spectra were vertically shifted for comparison purposes. The seawater 4.0 Gy (high Mg^2+^and SO_4_^2–^ concentrations) was prepared as suggested by Zaia.^47^.

Figure 2 presents the high-resolution XPS measurements around Fe 3p, O 1s, and C 1s. In Figure 2a (Fe 3p), the first three curves from the bottom refer to samples produced in ultrapure water. A broad peak with an asymmetric tail at the high-BE side is observed around 55 eV, typical of iron oxides.^51−54^ Such a broad peak results from various underlying physical phenomena, like electron exchange interaction, which produces multiplet splitting states, and electron correlation, which gives rise to several shake-ups and/or shake-off states.^55−60^ Fe 3p also contains peaks 3p_3/2_ and 3p_1/2_ due to spin–orbit splitting, which are not individually resolved since they are too close in energy and, therefore, beyond our energy resolution. Previous X-ray diffraction (XRD) measurements revealed that samples MGP and MGCN consisted of a single phase of magnetite (Fe_3_O_4_), while MGSCN also contained a significant amount of goethite (FeOOH).^12^ The center of the peaks for these three samples moves about +0.3 eV from MGP to MGSCN (see dashed straight lines). The full width at half-maximum (fwhm) for these three samples decreases from 2.90 eV (MGP) to 2.61 eV (MGCN) and then increases to 2.87 eV (MGSCN). Adding KCN and KSCN during the synthesis promotes a noticeable effect on the surface of the final compound. Considering that Fe_3_O_4_ is a mixed-valency oxide (Fe^2+^Fe_2_^+3^O_4_), the decrease of the fwhm and the displacement of the peak center to higher values of binding energy suggests that the additives promote the formation of a pure Fe(III) compound, like goethite or ferrihydrite. For the samples synthesized in seawater 4.0 Gy (top three curves), the Mg 2p peak is observed at the low-BE side of Fe 3p. Another peak observed at ∼ 64 eV (see black arrow) is associated with the well-known shakeup peak of Fe(III)-pure oxides.^51,59^

Figure 2b shows the O 1s region where we note significant differences among the three first curves from the bottom (samples synthesized in ultrapure water), especially for sample MGCN. O 1s region results from the convolution of several peaks coming from different oxygen functionalities, i. e., O–Fe, C–OH, O–C–O, C = O. The lowest BE peak is attributed to the lattice oxygen (O_latt._) of iron oxide (see black arrow). It has a smaller relative intensity than the other peaks, which are mainly associated with organic species. This is consistent with the survey spectrum, which shows a very high intensity of C 1s. Interestingly, for sample MGCN, the relative intensity of O_latt._ peak strongly increases, indicating that adding KCN favors the formation of iron oxide. Such a huge increase of O_latt._ peak intensity is not observed for MGSCN, although the spectral weight of this peak (O_latt._) is more significant than in the case of MGP. It is also worth noting that the overall width of the O 1s region for MGCN is smaller, suggesting that fewer oxygen species contribute to the peak or contribute less. The top three curves show the spectra of samples synthesized in seawater 4.0 Gy. All curves resemble the curve MGSCN, and when one looks carefully, curve MG4CN has a smaller width than MG4 and MG4SCN, as observed for the samples produced in ultrapure water.

Figure 2c presents the spectra of C 1s. The C 1s region was fitted following the procedure described in the literature.^35,42^ The inset shows a fit of the MGP curve as an example. The fwhm of the different functionalities ascribed to C–C/C–H (*E*_B_ = 284.8 eV), C–OH/C–O–C (*E*_B_ + ∼1.5 eV), C = O (*E*_B_ + ∼3.0 eV) and O–C = O (*E*_B_ + ∼4.0 eV) has been constrained to be the same during the fittings. The energy position of the main peak (lowest BE) was used for charge referencing purposes. All curves have a similar shape except for MGCN, where we identify peaks associated with K 2p_3/2_ and K 2p_1/2_. It is clear that the addition of KCN has a significant effect on the synthesized samples, especially in the case of ultrapure water.

It is important to remember that the surface sensitivity is different for Fe 3p, O 1s, and C 1s since the inelastic mean free path (IMFP) λ varies appreciably for these three elements. The calculated values of λ using the TPP2M model^60^ for an incident photon energy of 650 eV are approximately 1.1 nm for Fe 3p, 0.7 nm for O 1s, and 1.4 nm for C 1s. Since, on average, 95% of the photoelectron signal comes from a depth of 3λ, probing depths are 3.3 nm for Fe, 2.1 nm for O, and 4.2 nm for C. The largest surface sensitivity is obtained for O, which also has the highest photoionization cross-section.^61,62^

### 3.2 Fe L_23_-Edge Near-Edge X-ray Absorption Fine Structure (NEXAFS)

**Figure 3:**
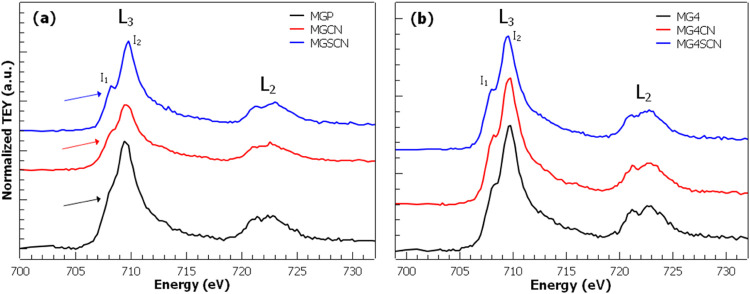
Fe L-edge NEXAFS measurements for samples synthesized in (a) ultrapure water and (b) seawater 4.0 Gy. Measurements were performed at room temperature in TEY mode. Spectra were vertically shifted for comparison purposes. The synthesis of MGCN/MG4CN and MGSCN/MG4SCN was carried out in the presence of 60 mmol of KCN and 60 mmol of KSCN, respectively. The seawater 4.0 Gy (high Mg^2+^and SO_4_^2–^ concentrations) was prepared as suggested by Zaia.^47^.

Figure 3 shows the Fe L-edge NEXAFS measurements performed at room temperature in TEY mode for samples synthesized in (a) ultrapure water and (b) seawater 4.0 Gy. The background was subtracted from all spectra by adjusting a straight line for the data in the pre-edge region, and normalization was done considering a linear function above the edge region using the Athena program.^59^ It has been shown that dipole-allowed 2p-3d transitions dominate the X-ray absorption process of iron oxides.^63−66^ There are two main spectral features present in the spectra: (i) due to Fe 2p spin–orbit coupling, the spectrum is divided into two well-resolved regions corresponding to L_3_ (706 – 713 eV) and L_2_ (719 – 726 eV) edges; (ii) an additional splitting is observed due to the crystal field, which lifts the degeneracy of the 3d levels and separates them in e_g_ and t_2g_ states. It is worth noticing that this crystal-field-induced splitting is strongly dependent on the coordination environment.^67,68^ L_3_ and L_2_ edges contain similar information, but our analysis will focus on L_3_ edge since it presents sharper and better-resolved features. Figure 3a shows the spectra for the samples synthesized in ultrapure water. All three spectra are similar except for the feature indicated by colored arrows (∼708 eV), which changes and becomes better resolved going from MGP to MGSCN. The spectrum for MGP sample is similar to Fe_3_O_4_ spectra published in the literature.^67,69−73^ Fe_3_O_4_ is a mixed-valence compound (Fe^2+^Fe_2_^3+^O_4_) with three different sites for Fe; 1/3 are Fe^2+^ ions octahedrally coordinated, 1/3 are Fe^3+^ ions octahedrally coordinated, and the remaining 1/3 are Fe^3+^ tetrahedrally coordinated. The overlap of states coming from distinct symmetries, e_g_/t_2g_ states for octahedral symmetry and *e*/*t*_2_ states for tetrahedral symmetry, and the mixed-valence character gives rise to poorly resolved peaks. One observes a clear change in the spectra with the addition of cyanide (KCN) and thiocyanate (KSCN), that is, the peaks become better resolved, especially for KSCN.

The spectra of samples synthesized in seawater 4.0 Gy shown in Figure 3b are similar when compared to each other, although there again, we can notice a change when KCN and KSCN are added. In the case of sample MG4P (without additives), the peaks at the L_3_ edge are again not so well-defined, becoming better resolved when KCN and KSCN are added. To obtain more insight into the effect of KCN and KSCN on the spectra, we measured the heights (*I*_1_ and *I*_2_) and energy separation (Δ) between the two peaks at the L_3_ edge. The energy positions of peaks I_1_ and I_2_ were obtained using the zero of the first derivative of the spectra. It has been previously shown that Δ is directly related to the ligand field splitting parameter 10Dq, which in turn is highly dependent on the coordination symmetry, while the intensity ratio (*R*_12_ = *I*_1_/*I*_2_) between *I*_1_ and *I*_2_ can be used to determine and quantify the Fe valence state.^74−76^ It has been observed that pure Fe(II) compounds present intensity ratios (*R*_12_) larger than 1.0 and energy splitting values (Δ) around 2.0 eV, while for pure Fe(III) compounds, *R*_12_ is close to 0.5 and Δ ≈ 1.5 eV. Magnetite, a mixed-valence compound, has an *R*_12_ value close to 0.7 and Δ < 1.5 eV.^70−72^Table 3 summarizes the values obtained for Δ and R_12_. We note that the sample synthesized in ultrapure water without any additive (MGP) is most likely magnetite. Adding KCN has roughly no effect on the values, although one observes a change in the shape of the curve. By adding KSCN Δ increases and *R*_12_ decreases, suggesting the formation of a pure Fe(III) environment like in goethite. In the case of samples synthesized in seawater 4.0 Gy, the values of Δ and *R*_12_ do not change and are those associated with iron oxides of pure Fe(III). Summing up, the NEXAFS data lead us to conclude that the surface of the sample MGP is most likely Fe_3_O_4_, and the addition of KCN and KSCN promotes the formation of compounds with Fe(III) in mostly octahedral coordination (Ex.: goethite, hematite, and ferrihydrite).

**Table 3:** Values of the Difference in Energy (Δ) of Peaks Labeled as *I*_1_ and *I*_2_ and Their Intensity Ratio (*R*_12_ = *I*_1_/*I*_2_)^a^^b^

Sample	Δ (eV)	*R*_12_
MGP	1.3	0.6
MGCN	1.2	0.6
MGSCN	1.4	0.5
MG4	1.5	0.5
MG4CN	1.5	0.5
MG4SCN	1.5	0.5

a As a measure of intensity, we used the peaks’ height.

b The synthesis of MGCN/MG4CN and MGSCN/MG4SCN was carried out in the presence of 60 mmol of KCN and 60 mmol of KSCN, respectively. The seawater 4.0 Gy (high Mg^2+^and SO_4_^2–^ concentrations) was prepared as suggested by Zaia.^47^

### 3.3 Transmission Electron Microscopy (TEM)

**Figure 4:**
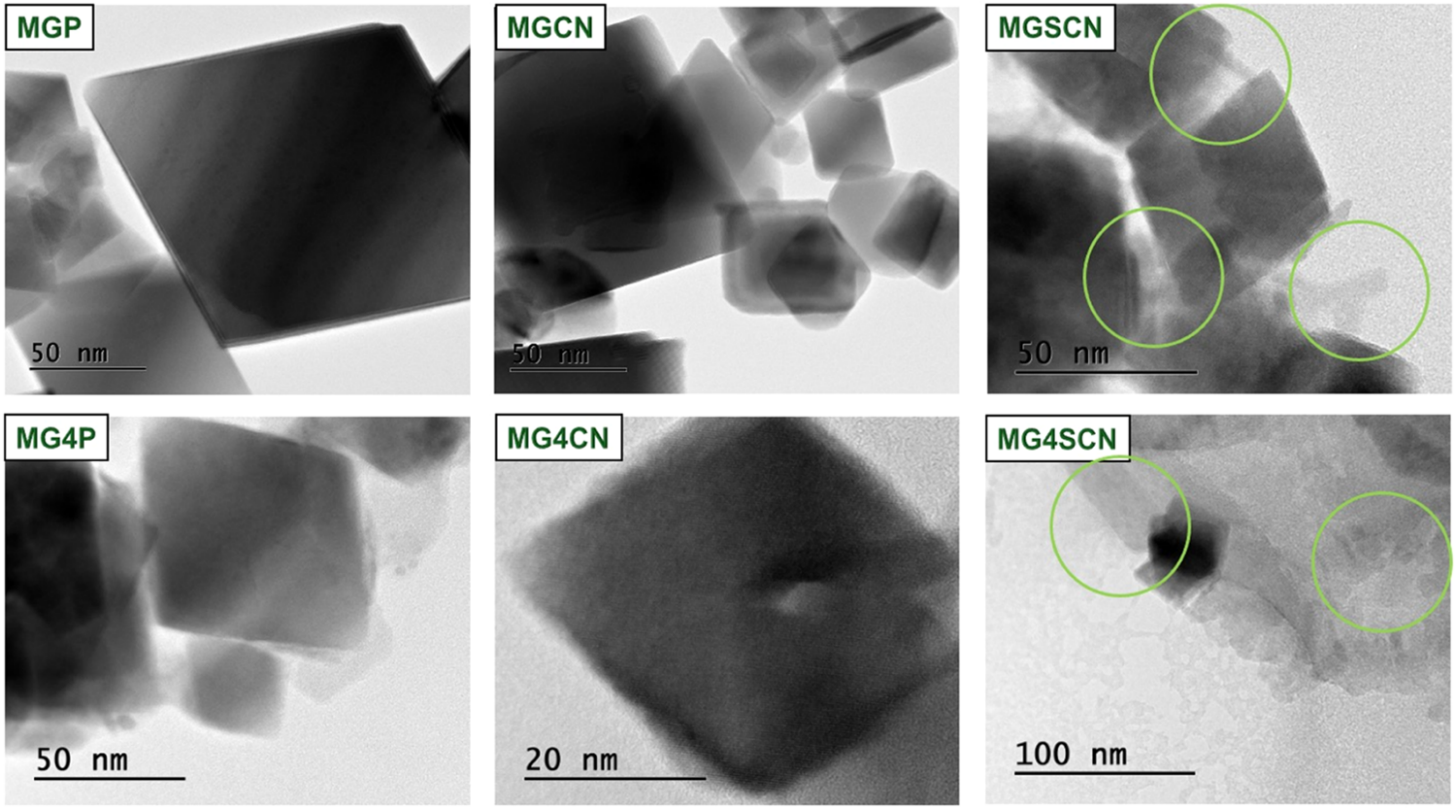
Transmission electron microscopy images of synthesized magnetite samples. (Green circles highlight rod-shaped gothite formation.) The synthesis of MGCN/MG4CN and MGSCN/MG4SCN was carried out in the presence of (60 mmol of KCN) and (60 mmol of KSCN), respectively. The seawater 4.0 Gy (high Mg^2+^ and SO_4_^2–^ concentrations) was prepared as suggested by Zaia.^47^.

Figure 4 shows TEM images of magnetite samples at different amplifications. The MGP sample demonstrates typical magnetite particles’ octahedral shape.^12,76−81^ The same overall octahedral shape was observed for the MGCN sample, showing that cyanide ions have a minor effect on the magnetite formation mechanism. In the case of MGSCN sample, TEM image shows clear changes. There is still evidence for the formation of magnetite, but one also perceives small rod-shaped regions, which are characteristic of goethite samples.^12^ Structures with low crystallinity are also visualized, possibly associated with the formation of ferrihydrite. The image of the MG4SCN sample shows a decrease in the amount of magnetite crystals formed and one also observes important amorphous areas referring to the formation of ferrihydrite.

Regarding samples synthesized with SCN ions, the goethite and ferrihydrite phases are formed separately from the magnetite crystals. This is not what happens with the MG4P and MG4CN samples. In these samples, the magnetite crystals do not appear as well formed as in samples synthesized in ultrapure water with heterogeneous octahedron faces. It is also possible to observe regions with amorphous areas, possibly associated with the formation of ferrihydrite. The microscopy images agree with the XPS findings and corroborate previously published work with Mössbauer and XRD analyses.^12^

### 3.4 Magnetization Measurements

**Figure 5:**
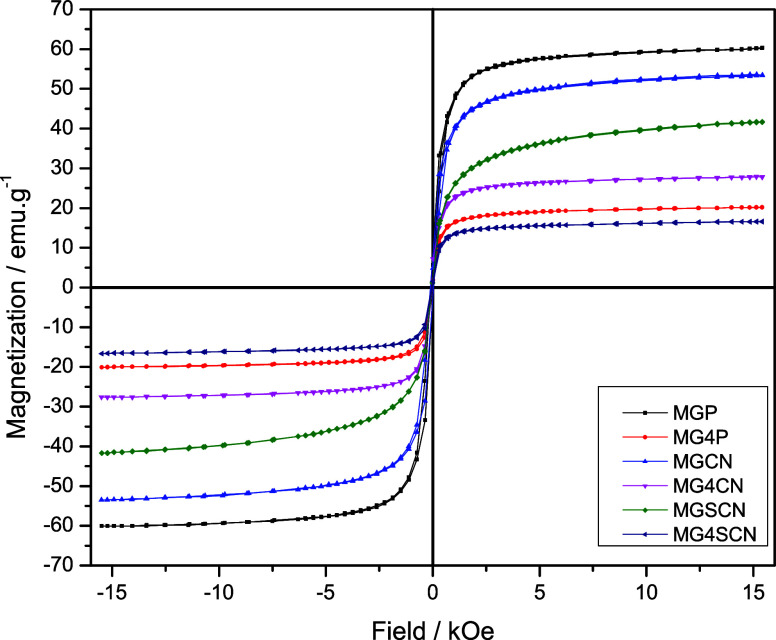
Room-temperature magnetization curves of magnetite samples. The synthesis of MGCN/MG4CN and MGSCN/MG4SCN was carried out in the presence of 60 mmol of KCN and 60 mmol of KSCN, respectively. The seawater 4.0 Gy (high Mg^2+^ and SO_4_^2–^ concentrations) was prepared as suggested by Zaia.^47^.

Figure 5 shows the room-temperature magnetization curves for all samples. We observe two clear differences between the two sets of samples: (i) the saturation magnetization (*M*_s_) is roughly 2 times higher for samples synthesized in ultrapure water, in comparison to samples produced in seawater 4.0 Gy and (ii) while for samples produced in seawater 4.0 Gy saturation is mostly attained at ∼5 kOe, for samples synthesized in ultrapure water it is much higher, above 10 kOe. M_s_ for MGP sample (∼60 emu/g) falls within the range of values reported in the literature for nanosized magnetite.^76−81^ In the case of samples produced in ultrapure water, the addition of KCN and KSCN leads to a decrease of *M*_s_. Such decrease is consistent with the formation of goethite/ferrihydrite phases observed in XPS and NEXAFS data, which do not present substantial magnetic susceptibility.^81^ Samples synthesized in seawater show a drastic reduction in magnetization, consistent with the observations of NEXAFS data that show the presence of phases with very low magnetic susceptibility, such as goethite and ferrihydrite. The MG4SCN sample presents the lowest *M*_s_, which is in agreement with TEM images that show a smaller presence of magnetite crystals. Table 4 presents the values of *M*_s_ for all samples.

**Table 4:** Saturation Magnetization (*M*_s_) Values of the Magnetite Samples^a^

Sample	*M*_s_ (emu/g)
MGP	59.6
MG4P	20.6
MGCN	53.6
MG4CN	27.6
MGSCN	41.6
MG4SCN	16.8

a The synthesis of MGCN/MG4CN and MGSCN/MG4SCN was carried out in the presence of 60 mmol of KCN and 60 mmol of KSCN, respectively. The seawater 4.0 Gy (high Mg^2+^ and SO_4_^2–^ concentrations) was prepared as suggested by Zaia.^47^

## 4 Relevance to Prebiotic Chemistry

Several studies confirm Bernal’s hypothesis that minerals could have played important roles in the origin of life, such as preconcentrating biomolecules and biomolecule precursors from dilute solutions, catalyzing the formation of polymers and protecting molecules against degradation by radiation or hydrolysis.^11,13,47,79−87^ In the present work, magnetite was synthesized in the presence of cyanide, thiocyanate, and artificial seawater, and the materials were characterized by X-ray photoelectron spectroscopy (XPS), Fe L_23_ near-edge X-ray absorption fine structure (NEXAFS), transmission electron microscopy (TEM), and magnetization measurements. Minerals are complex substances for which a thorough interpretation is a hard task. Therefore, making use of various characterization techniques covering different properties turns out to be very important to get a more precise understanding of its physicochemical properties. When one aims to investigate the origins of the basic building blocks leading to different life forms on Earth it would be more interesting to use natural minerals than synthetic ones for prebiotic chemistry experiments since the latter will hardly reproduce the complexity of its natural counterpart. The complexity of the natural minerals could be reduced by washing them with acid or basic solutions or even adsorbing metals on them. These are common procedures for clay minerals used in prebiotic experiments.^81,82^ However, even synthetic minerals could show small differences depending on the synthesis route.^84^ Thus, we should ask ourselves, how deep should we go in characterizing a mineral? And are small differences important for prebiotic chemistry experiments? This topic has already been discussed elsewhere.^47^ Goethite synthesized by two different routes showed different electronic paramagnetic spectra,^86^ and these goetithes had different effects on the polymerization of amino acids.^85^ We were able to show that XPS and NEXAFS data analysis are in line with data from X-ray diffractometry and Mössbauer spectroscopy.^12^ XPS and NEXAFS analysis of the materials were especially important because they revealed that differences on the surface are observed depending on the magnetite synthesis route. Information about the surface chemistry of minerals is fundamental since, in most cases, molecules’ adsorption, polymerization, and protection occur at the surface level.^10,11,13,47,83−87^ This information is of great value in interpreting data from prebiotic chemistry experiments.

In the context of the primitive Earth, magnetite may have functioned as a crucial catalytic surface for polymerization reactions of amino acids and nucleotides, promoting the formation of complex biomolecules.^88^ The results presented indicate that the presence of cyanide stabilizes magnetite, preventing its oxidation to Fe^3+^ phases such as goethite and ferrihydrite. Cyanide is known to form highly stable complexes with iron, mainly with Fe^2+^ and Fe^3+^, due to its π acceptor potential (backdonation), depending on the redox potential of the medium. These coordination bonds can modulate the rate of iron oxidation and influence mineral precipitation. The main factors that favor magnetite in the presence of cyanide are (i) Stabilization of Fe^2+^ in solution. Cyanide binds strongly to Fe^2+^, forming complexes that have low oxidative reactivity. This reduces the spontaneous conversion of Fe^2+^ to Fe^3+^, preventing the formation of amorphous iron hydroxides and favoring the precipitation of magnetite, which contains both Fe^2+^ and Fe^3+^;^89^ (ii) Control of oxidation kinetics. Magnetite forms under conditions where Fe^2+^ is partially oxidized, but without Fe^3+^ becoming the dominant species. Cyanide acts as a stabilizing agent, reducing the availability of free Fe^3+^ and preventing the formation of phases such as goethite and ferrihydrite, which require further oxidation;^90^ (iii) Reduction of secondary phase growth. In cyanide-free syntheses, highly solvated Fe^3+^ species favor the formation of hydroxides such as ferrihydrite. However, cyanide can inhibit this nucleation by forming soluble complexes that keep Fe^3+^ in solution for longer, allowing magnetite to grow in a more controlled and homogeneous manner.^91^

Thiocyanate (SCN^–^), on the other hand, being a typical σ/π donor ligand, exhibits a distinct behavior in the iron-aqueous system. The main effects of thiocyanate are as follows: (i) Facilitated oxidation of Fe^2+^ to Fe^3+^. SCN^–^ does not form such stable complexes with Fe^2+^ as CN^–^. This means that Fe^2+^ becomes more available for oxidation under aerobic conditions, leading to the predominant formation of Fe^3+^ and precipitation of phases containing only Fe^3+^, such as goethite and ferrihydrite.^92^ (ii) Catalytic effect of SCN^–^ on Fe^3+^ hydrolysis. Unlike CN^–^, which sequesters Fe^3+^ in solution, SCN^–^ can participate in reactions that increase the rate of Fe^3+^ hydrolysis, leading to the formation of hydroxylated intermediates that rapidly crystallize in forms such as goethite (α-FeOOH) and ferrihydrite (Fe_5_HO_8_ 4H_2_O);^89^ (iii) Role of salinity and additional ions. In solutions containing SCN^–^ and high concentrations of Na^+^, Cl^–^ and SO_4_^2–^ (as in primitive ocean water), the precipitation of Fe^3+^ hydroxides are favored, as these ions compete with SCN^–^ for complexation sites on Fe^3+^, reducing its solubility and accelerating the nucleation of pure Fe^3+^ phases.^93^ These mechanisms show that the formation of different mineral phases in the experiment is directly related to the specific interactions of the added anions with the iron ions in solution. Cyanide favors magnetite by stabilizing Fe^2+^ and delaying its oxidation, while thiocyanate promotes goethite and ferrihydrite by facilitating Fe^2+^ oxidation and stimulating Fe^3+^ hydrolysis. This selectivity has important implications for both the study of early Earth mineralogy and technological applications, such as the controlled synthesis of iron oxides for use in environmental remediation and catalysis.

The selective formation of magnetite, goethite, and ferrihydrite under simulated early Earth conditions suggests that iron mineralogy may have been a critical factor in the evolution of prebiotic reactions. Stabilization of magnetite by the presence of cyanide could have favored longer-lasting catalytic surfaces, while partial conversion to goethite and ferrihydrite in the presence of thiocyanate may indicate the existence of multiple geochemical microenvironments where different prebiotic reactions occurred. These findings reinforce the hypothesis that iron minerals not only provided surfaces for adsorption and polymerization of biomolecules, but also directly influenced the availability and chemical stability of reactants on the early Earth.^94,95^ Furthermore, this study highlights the need to consider the complexity of geochemical processes in experimental modeling of the origin of life, ensuring that environmental conditions are realistic and represent prebiotic evolution scenarios. Schwartz^96^ argues that prebiotic processes tend to generate complex and chaotic mixtures, hindering chemical and biological evolution, and that minerals could play a role in chemical organization, but notes that many experiments fail to demonstrate selectivity. This study suggests that minerals and primitive molecules evolved synergistically, with some minerals showing selectivity for some molecules and some molecules inducing the formation of other minerals. Thus, minerals and ions could have played critical roles in chemical selection and organization, creating more favorable microenvironments to the origin of life.

## 5 Conclusions

Characterizations of magnetite samples synthesized under prebiotic conditions revealed significant variations in elemental composition when different ions were added during synthesis. Including cyanide and thiocyanate notably influenced the formation of iron oxide compounds, producing compounds with oxidized iron such as goethite or ferrihydrite. XPS and NEXAFS data clearly show surface transformations where iron is predominantly located in octahedral coordination environments upon adding KCN, KSCN, and ions from pristine seawater.

TEM imaging showed typical octahedral-shaped magnetite particles for samples synthesized in ultrapure water. The images suggest that cyanide protects the magnetite formation mechanism, as observed in XPS. In contrast, samples synthesized in seawater and added KSCN showed a mixture of magnetite, goethite, and ferrihydrite phases, consistent with the XPS and NEXAFS results. Saturation magnetization decreases when KCN and KSCN is added, which is particularly pronounced in samples synthesized in seawater. Such decrease is the result of the formation of phases with smaller magnetic susceptibility, such as goethite and ferrihydrite.

The analyses highlight the interaction between the synthesis conditions, the chemical composition, and the properties of the resulting material. These discoveries deepen our understanding of Earth’s earliest geochemical processes and have implications for fields ranging from environmental science to materials synthesis.

The mineral phases obtained in the presence of cyanide and thiocyanate can be attributed to their distinct coordination interactions with iron ions. Because cyanide ion (CN^–^) forms very stable complexes with Fe^2+^, then slows down its oxidation to Fe^3+^. This stable complex favors the redox balance necessary for the synthesis of magnetite.^97,98^ Also, this stabilization of Fe^2+^ allows the nucleation and growth process to occur in a more orderly manner, promoting the formation of magnetite crystals with a well-defined structure. In contrast, because thiocyanate (SCN^–^) presents weak coordination with Fe^2+^, directing the system toward the formation of oxidized phases, such as goethite and ferrihydrite. In addition, the presence of Cl^–^ and SO_4_^2–^, from the artificial seawater, can further modulate the nucleation and growth processes, altering the surface energy of the forming nuclei and, consequently, the morphology and distribution of the resulting phases. Thus, small variations in ionic composition and coordination mechanisms may have played a critical role in the mineralogical diversity observed on the primitive Earth, directly influencing the geochemical niches available for prebiotic reactions.

The stabilization of magnetite by cyanide and the partial conversion to goethite and ferrihydrite by thiocyanate indicate that iron minerals provided different surfaces for adsorption and polymerization of biomolecules. The work of Schwartz argues that prebiotic processes tend to generate complex and chaotic mixtures, hindering chemical and biological evolution. Besides, minerals could play a role in the molecular evolution (adsorption and polymerization of biomolecules), but many experiments fail to demonstrate selectivity. Perhaps, as demonstrated in this study, minerals and primitive molecules evolved in synergy, in a two-way road, with some minerals showing selectivity for some molecules and some molecules inducing the formation of other minerals.

Also, the results obtained herein can be applied in environmental studies to understand the dispersion of contaminants in industrial areas and regions impacted by acidification and acid mine drainage processes, where iron and sulfur often interact to form secondary phases of iron oxides and hydroxides.^99^ Furthermore, the variation in magnetic susceptibility of the formed phases can provide clues about the evolution of iron-rich sedimentary deposits and their influence on past biogeochemical cycles.^100^ Thus, our findings reinforce the importance of considering mineral precipitation processes to assess the evolution of geochemical environments at scales ranging from the early Earth to modern contamination scenarios.

These results also have significant implications for materials science, especially in the controlled synthesis of iron oxides with specific properties. The influence of ions on the formation of different iron phases can be exploited for the development of new materials with applications in catalysis, sensors and environmental remediation. Magnetite, for example, is widely used in the fabrication of magnetic nanomaterials for biomedical and electronic applications and understanding the factors that control its stability, and chemical purity is essential to optimize these processes.^90^ Furthermore, the preferential conversion of magnetite to goethite and ferrihydrite in salt-rich media may be relevant for the development of surface modification strategies in applications such as heavy metal adsorption and removal of organic contaminants from water.^99^ From these findings, the manipulation of synthetic chemistry can be applied to produce iron oxides with different morphologies and magnetic properties, allowing the customization of materials for specific needs of industry and materials science.^101^
